# Remarks upon the Question, Why Has the Application of a Temporary Ligature, upon the Principal Artery of a Limb, for the Cure of Aneurism, Been Sometimes Considered Insufficient to Cause an Obliteration of the Vessel?

**Published:** 1830-08

**Authors:** Ant. Scarpa


### TEMPORARY LIGATURE.

*Remarks upon the Question, Why has the Application of a temporary Ligature, upon the principal Artery of a Limb, for the Cure of Aneurism, been sometimes considered insufficient to cause an Obliteration of the Vessel?* By Professor ANT. SCARPA.*

IN various parts of my treatise upon the Application of Ligatures to the principal Arteries of the Limbs, and in the appendix to my work on Aneurism, and particularly in my practical reflections upon the ligature of the principal arteries of the limbs, according to the method of HUNTER, I have proved, by facts from dissection and practice, that, in general, a ligature upon the principal artery of a limb, although it may completely obliterate that vessel, cannot at the same time cause obliteration of the anastamosing branches which exist between the lateral vessels and the principal trunk, from the ligature to the aneurismal sac, nor the obliteration of the collateral arteries which open directly into the sac. As the size of the anastomosing branches varies greatly in different individuals, it necessarily follows that the phenomena which succeed the application of the ligature also vary in different subjects after the operation, and that the return of the circulation of the blood in that portion of the artery comprised between the ligature and the aneurism may be more or less apparent. But this effect, which might at first lead to the opinion that the ligature was insufficient, does not prevent the ulterior cure of the disease. I have thus previously expressed my opinion upon this subject:† "It is said that, after the principal artery of a limb has been tied, it is possible that the blood may flow into the aneurismal sac by the anastomosing branches, and that the operation may thus be useless. Far from denying the possibility of the phenomenon, I believe, on the contrary, that it always exists, in a greater or lesser degree in different individuals, after the application of the ligature according to Hunter's method, but that the success of the operation is not interfered with. This is easily comprehended, if we reflect that the blood enters the tumor but in a very small quantity, and that it has not that impulsive force which is necessary to distend the sac, so that the slowness of its current favors the deposition of fibrous laminæ in the interior of the sac, the cavity of which is gradually filled and obliterated. This sanguineous mass

* Annali Univ. di Med. Janv. 1830.

† Opus. di Chirurgia, vol. ii. p. 121. Pavie, 1825.

is afterwards gradually absorbed, and the tumor is at length reduced to a very small volume."

The following case corroborates my opinion. A man, fifty years of age, had popliteal aneurism, for which the femoral artery was tied in the upper third of the thigh. The ligature was removed on the third day. From the moment the vessel was secured, the aneurismal tumor diminished in size, and ceased to pulsate. The day after the removal of the ligature, obscure and deep-seated pulsation in the tumor was perceived; but its volume was not increased, it remained of the same size as after the operation. On the following day, the tumor gradually diminished, and became harder; the pulsations became weaker, and insensibly disappeared, and the patient was perfectly cured.

In cases in which a temporary reappearance of pulsation in the aneurismal sac occurs, the pulsation is always feeble and obscure; the size of the tumor does not increase, but it continues of the same magnitude as immediately after the operation, and after the removal of the ligature gradually diminishes. But, when the operation has not produced the desired result, the obliteration of the artery, the blood continues to enter the sac with great force and rapidity, and thus distends it. An attentive examination of the tumor in these two cases, will prevent us from confounding them. In the last particularly, compression of the vessel above the tumor suspends all pulsation in it, and the pulsation returns when the compression is removed. When the *temporary* ligature has obliterated the artery, the diminished volume of the tumor coutinues after the removal of the ligature; and, if subsequently obscure pulsation is observed in it, it is not until after the lapse of days, weeks, or even months. This variation in the period at which pulsation in the tumor occurs results from the more or less rapid dilatation of the anastomosing branches in different subjects. When aneurism of the femoral artery is seated near the groin, a little below the origin of the profunda, it is necessary to apply a ligature upon the external iliac above the epigastric and circumflex arteries. Now, in this case, these two arteries become, from their communication with the branches supplying the parietes of the thorax and abdomen, two anastomosing vessels, which gradually transmit a more or less abundant quantity of blood into the principal trunk below the ligature. Here we might have the greatest apprehension of failure from the operation; but experience proves its success: for, although the epigastric and circumflex arteries rapidly and considerably dilate, yet the current of blood in them has no longer the same velocity, nor does it enter the aneurismal sac with so much force. Besides, the blood, transmitted by these two arteries into the femoral trunk, finding a free and ready issue by the profunda, the greatest part of it follows this track, whilst a very small quantity penetrates the sac, and may give rise to very obscure pulsations in it. This slow deposition of blood in the tumor is favorable to the ulterior obliteration of the cavity. Since experience demonstrates most positively, that when the aneurismal tumor remains, after the removal of the ligature, of the same size to which it had been reduced by the operation, that the obliteration of the artery is not the less certain because obscure and deep-seated pulsations are detected: it is clear that it is perfectly useless to introduce a finger into the wound, for the purpose of ascertaining whether the blood enters the vessel below the point at which it had been secured.

The following cases, related by different surgeons, corroborate the preceding opinions.

Case I. Demonti André, æt. forty, while carrying upon his shoulder a heavy and pointed sithe, stooped down to the ground: the sithe fell from his shoulder, and pierced the calf of his right leg. A stream of blood immediately flowed from the wound, and the man restrained the hemorrhage by pressure. He was carried to the hospital, and M. Kruch examined the wound. There was now no bleeding. Adhesive plaster and cold lotions were applied, and perfect rest was ordered. For nine days no particular symptom occurred, and the same treatment was continued; when the patient, in stepping hastily from his bed, immediately perceived, above the cicatrix, a hard circumscribed tumor, presenting synchronous pulsations with the radial artery, which pulsations disappeared when the femoral artery was pressed. It was evident that there was aneurism of the posterior tibial artery, and consequently a ligature was applied upon the femoral artery in the upper third of the thigh, by M. Kruch, on the 21st of August. Immediately after the operation, the pulsations in the tumor ceased, and its size was much diminished. The limb was surrounded with bladders filled with hot water.

The ligature was removed on the third day: there was not the slightest pulsation in the tumor. Wound looked healthy.

About five weeks after the operation, the wound was completely healed, and there was now observed in the tumor (the size of which was much diminished) very distinct, yet deep-seated and feeble, pulsations. They were more evident when the centre of the tumor was compressed. The weakness of these pulsations, which were not attended by the slightest increase in the size of the tumor, induced Dr. Kruch to believe that they arose from some anastomosing branches which entered the trunk of the femoral artery below the ligature, or the aneurismal sac itself, and not from the entrance of the blood directly through the artery which had been tied. Acting upon this opinion, the only treatment adapted was the application of cold astringent lotions, to assist the efforts of nature in the obliteration of the aneurismal sac. The tumor gradually became harder, the pulsations in it less and less, and the patient was perfectly cured. On the 12th of October, he rose without any other inconvenience than weakness in the affected limb, upon which a bandage was applied, with directions that it might be worn for some time, when he left the hospital.

This case of aneurism of the posterior tibial artery, analogous to several others that have been published by myself and other practitioners, clearly proves that a ligature applied to the principal arterial trunk of a limb not only cures aneurism of *it,* but also that of any of its principal divisions. It is especially in such cases as the above, where the wounded artery is deep-seated, that the improvement of surgery is manifest; for the practice adopted is much more advantageous than that of exposing the vessel in the midst of parts which are frequently in a state of gangrene, and tying it, with great difficulty, above and below the wound. It must be observed, that the success of applying a ligature upon the principal trunk, when one of its branches is affected with aneurism, is the more certain in proportion as the wound of the branch is near the union with the artery which is tied.

Case II. A. Giacomelli, æt. nineteen, of robust constitution, had the left brachial artery wounded in bleeding, in March 1829. Violent hemorrhage instantly occurred, and was arrested by a tight bandage. The pressure was continued for a month, and produced great inflammation. When the swelling of the limb subsided, an indolent pulsating tumor was perceived, exactly at the spot where the operation of venesection had been performed. As the size of this tumor daily increased, the patient presented himself at the hospital of Pavia, four months after the accident.

Professor Cairoli found, at the bend of the arm, a swelling about the size of a hen's egg, which pulsated synchronously with the artery at the wrist, and had every appearance of varicose aneurism. A ligature was applied above the tumor, the 17th of July, by Mr. C. As soon as the artery was tied, the pulsations ceased in the tumor, and it diminished to about three fourths of its former size. The various phenomena which commonly succeed the application of a ligature upon the principal artery of a limb were observed. The arm was surrounded with bladders filled with hot water, and moistened from time to time with an emollient and warm decoction. On the third day, feeble pulsations were detected in the ulnar artery. On the evening of the 20th, (three days and a half after the operation,) Professor C., remarking that the tumor still continued to lessen in size, and that there was not the slightest appearance of pulsation in it, or below the point where the artery had been tied, divided the ligature, and removed it. No new symptoms followed.

The following night, the patient was restless; he felt much heat and pain in the forearm.

21st.—High fever. Pulsations in the ulnar and radial artery as strong as in the natural state; slight pulsations in the tumor, but no increase of its size; wound much inflamed, and the brachial artery immediately below the ligature pulsated violently. As the tumor had not increased in size, M. Cairoli observed to the pupils, that the pulsations in it, and in the brachial artery, arose entirely from the return of blood by the collateral branches, and not from the non-obliteration of the brachial artery where it had been tied. The issue of the case fully confirmed this opinion. The inflammatory symptoms were actively treated by antiphlogistic means, and emollient fomentations around the limb; the tumor was covered with iced water, and continued to decrease in size. By the 4th of August, (fourteen days after the removal of the ligature,) all the pulsations had entirely ceased. The wound soon healed, and the patient left the hospital perfectly cured.

Case III. Nicolas Garolfi, æt. thirty, was admitted into the hospital of Plaisance, to be operated upon for popliteal aneurism. The tumor was the size of a fist, and situated in the hollow of the right ham. Professor Morigi tied the femoral artery, the 28th of January, 1824. The pulsation ceased immediately in the tumor, as well as the pains which had been before felt in it.

February 1st.—Ligature removed. The tumor was diminished to about one half of its former size. The foot was warm, and but little sensible to the touch; the extremities of the toes were cold.

The tenth day, the wound had nearly cicatrised; the circulation was partly re-established in the lower part of the limb, which had recovered its natural warmth and sensibility. The leg could be bent upon the thigh without much difficulty.

Twentieth day.—State of the patient so satisfactory, that he was permitted to walk on crutches, the limb being bandaged.

Three days from the commencement of his taking exercise, feeble and deep-seated pulsations were felt in the tumor: perfect rest was consequently enjoined, and the tumor was covered with cold lotions. A most careful examination of the course of the femoral artery below the ligature was made, but not the slightest pulsation was detected. The dull and deep pulsation in the tumor continued for four days, but it did not increase in size. This pulsation insensibly diminished, and the patient rapidly recovered, and left the hospital perfectly cured fifty-six days after the operation.

Two years afterwards, the same person again appeared at the hospital, with popliteal aneurism of the left leg. The limb was much swollen, and therefore Professor Morigi deemed some delay prudent. After a few days' rest, the operation was performed, as it had been in the other extremity, with complete success. The cure was perfect in forty-three days. Three years have now elapsed, and the man enjoys good health.

It will have been observed, that in this patient the circulation, which was for a short time re-established in the sac by the anastomosing branches of the right femoral artery, was only manifested by weak and deep-seated pulsations in the sac, which occurred twenty-three days after the operation. It is evident that this phenomenon arose from the too early exercise in which the patient indulged. After the operation upon the left limb, no such symptom occurred, probably because the anastomosing branches were not so largely dilated on this side, and probably also because, during the whole course of the treatment, the patient remained perfectly quiet. It must, then, be from an unjust prejudice, or an absolute ignorance of the facts which are demonstrated by physiology and practical surgery, that the opinion is maintained that the treatment by the *temporary* ligature is more likely to be followed by a return of the disease, because pulsations may occur in the aneurismal sac some time after the removal of the ligature. It is also strange that this phenomenon should be attributed to the *temporary* ligature, inasmuch as it is just as likely to occur when the *permanent* ligature has been applied, after it has been spontaneously separated from the vessel, and discharged or removed from the wound. I could cite many examples of this fact, but the following will be sufficient: it was communicated by Dr. MONTHEUTH to Mr. WISHART, professor of surgery at Edinburgh.

A person had in the ham an aneurismal tumor, the size of an orange. Dr. M. performed Hunter's operation. Thirteen days after the operation, the ligature spontaneously separated itself from the artery. The tumor, which had been suddenly reduced in size after the application of the ligature, continued to diminish until it was no larger than a chesnut. The wound in the thigh healed. Three months after, the patient complained of feeling a pulsation in the tumor which corresponded with that of the arteries, but weak and deep-seated; the tumor did not increase in size. Dr. M., who was convinced that the femoral artery was perfectly obliterated, first employed a compressing bandage over the whole limb, to remove the pulsation, and to accelerate the disappearance of the tumor; but this treatment was unsuccessful. Attempts were then made to compress the tumor alone by means of a cushion bandage, but the patient could not bear it more than half an hour. Notwithstanding the short time it was applied, Dr. M. was not surprised to find the pulsation gone when he removed the bandage. The centre of the tumor no longer indicated the presence of fluid: it was hard and solid. From this time absorption of the coagulum rapidly took place, and the patient was perfectly cured.

The anatomical and pathological remarks which I have offered appear to me important from their practical utility, and the improvements they suggest in the practice recommended by Hunter for the cure of external aneurisms. I am persuaded that it has not unfrequently happened, where the *temporary* ligature has been applied to the brachial or femoral artery, that mistakes have arisen as to the true cause of the phenomena which I have just mentioned; and that, by confounding the pulsations which arise, at an uncertain period, after the removal of the ligature, from the presence of the collateral vessels, with those which depend upon the non-obliteration of the arterial trunk upon which the ligature has been placed, that a second ligature has been applied above the first, and that thus the patient has been unnecessarily subjected to another operation.

---

The principal object of the above paper is to point out the unimportance of certain symptoms which sometimes occur after the application of the *temporary* ligature, and which have not seldom been supposed to prove the inefficacy of such a mode of practice. We regret, however, that the celebrated author has not pointed out the advantages obtained in the cases he relates, by the use of the *temporary* instead of the *permanent* ligature. Has he not adverted to this point because he no longer thinks so highly of this mode of operating; or does he admit that the advantage of the *temporary* ligature is not well illustrated in the instances he relates? The details of the cases are not sufficient to enable us to judge of all the points of comparison between the two plans; but to one point we may refer, namely, the *time* required for the healing of the wound, and the period at which the patients were discharged from the hospital. In the first case, the ligature was removed from the femoral artery on the third day. In five weeks the wound was nearly healed; the patient left the hospital fifty-two days after the operation. In the second case, the ligature was removed from the brachial artery three days and a half after its application: fourteen days after this, it is reported that the pulsations in the tumor had entirely ceased, and it is added, "the wound now healed, and the patient left the hospital cured." In the third case, the ligature was taken from the femoral artery four days after it had been applied: on the tenth day the wound had nearly cicatrised; the patient quitted the hospital fifty-six days after the operation. In the fourth case, one of femoral aneurism, the cure was perfected in forty-three days. There was probably, then, little gained as to *time* in these cases by the use of the temporary ligature. Of its superior safety, and advantages in other respects, the facts are not yet sufficient to enable us to form an opinion. We refer our readers for a concise yet interesting account of the opinions and experiments of various surgeons relative to the use of the *temporary* ligature, to COOPER'S Surgical Dictionary, p. 131, sixth edition. Mr. Cooper is adverse to the practice: his mind "has long been made up about the inexpediency of the temporary ligature as an innovation in surgery."*

* Mr. Guthrie, in his recent work on Diseases and Injuries of the Arteries has not stated his opinion respecting the use of the temporary ligature, but we know that he objects to its employment under any circumstances.—EDITOR.

